# Debriefing about the challenges of working in a remote area: A qualitative study of Australian allied health professionals’ perspectives on clinical supervision

**DOI:** 10.1371/journal.pone.0213613

**Published:** 2019-03-14

**Authors:** Priya Martin, Saravana Kumar, Lucylynn Lizarondo, Katherine Baldock

**Affiliations:** 1 School of Health Sciences, University of South Australia, Adelaide, South Australia, Australia; 2 Cunningham Centre, Darling Downs Hospital and Health Service, Toowoomba, Queensland, Australia; 3 Joanna Briggs Institute, University of Adelaide, Adelaide, Australia; Hofstra University, UNITED STATES

## Abstract

**Background:**

The benefits of clinical supervision are more pronounced for health professionals in rural and remote areas. Most clinical supervision studies to date have occurred in metropolitan centres and have used the survey methodology to capture participant experiences. There is a lack of qualitative research that captures participants’ lived experiences with clinical supervision at the frontline.

**Methods:**

Participants were recruited from rural and remote sites of two Australian states using a purposive maximum variation sampling strategy. Data were collected through individual, semi-structured interviews with participants. Data were analysed using content analysis and themes were developed. Sixteen participants from six professions completed the interviews.

**Results:**

Eight themes were developed including the content of supervision, context of supervision, value of supervision, increased need for professional support and unique characteristics of rural and remote clinical supervision.

**Conclusions:**

This study has highlighted the value of clinical supervision for the rural and remote health professional workforce. Furthermore, it has shed light on the unique characteristics of clinical supervision in this population. This information can be used by organisations and health professionals to ensure clinical supervision partnerships are effective thereby enhancing rural and remote workforce recruitment and retention.

## Introduction

Clinical supervision [CS] is a form of work-related support for health professionals which ensures that healthcare delivery is safe and of high quality [1,2]. Although, there are various definitions of CS and varied understanding of the concept, this study utilized the CS definition proposed by Milne [3] as ‘the formal provision, by approved supervisors, of relationship-based education and training that is work-focused and which manages, supports, develops and evaluates the work of colleague/s’. The rationale for using this definition is that the study described in this paper pertains to a one-to-one relationship between two post-registration health professionals, that not only serves a clinical governance purpose but also provides ongoing support to enhance the skills, knowledge and professional development of the supervisee. Information on the benefits of CS to patients, health professionals and organisations is well-documented in the literature. The benefits include enhanced safety and quality of patient care, improved workforce retention and a more supported and satisfied workforce. Although the benefits of CS are well-accepted, research, especially qualitative, in this area is lacking [1, 4–8].

Proctor’s model of CS is a widely used and reported model [9, 10]. This model consists of three domains: normative or managerial [e.g., promoting and complying with policies and procedures]; formative or educational [e.g., knowledge and skill development]; and restorative or pastoral support [e.g., understanding and managing emotional wellbeing]. It can be suggested that these three domains also explore quality elements of CS including technical aspects [such as effectiveness as measured through formative functions] and functional aspects [such as expectations and support as measured through normative and restorative functions].

The importance of CS is more pronounced in countries with a geographically dispersed population such as Australia [11–14]. Health professionals working in rural and remote locations in particular often experience professional isolation [14]. Health organisations use CS as one means of improving professional support of these staff. Whilst this is common practice [15], there is a lack of research on what happens at the frontline [12, 14]. This is especially true for allied health professionals as most CS literature to date has focused on nurses and mental health professions [16, 17]. As rural and remote practice comes with unique challenges [14], such as generalist roles with a broad scope of practice, it warrants further investigation.

Most existing studies have used surveys to investigate CS practices [5, 11, 12, 16, 18, 19]. Whilst survey studies are useful in building the evidence base, it is equally important to understand individual clinician perspectives to explain and contextualize survey findings. Studies are required that capture lived experiences of clinicians to understand what happens at the coal face. For example, an Australian survey study [12] with 159 rural and remote allied health professionals identified various factors that contributed to high quality supervision such as supervisee’s work setting, nature of job, supervisor and supervisee age, practice area, time in work role and CS frequency. Whilst the survey study identified the factors that contribute high quality CS, it did not explore in-depth the CS experiences of these professionals.

Some CS studies reported in the literature have used qualitative methodologies including individual interviews. For example, an Australian study [13] explored occupational therapist perspectives of CS in a qualitative study using individual interviews. The findings of this study provided rich information on the importance of the supervisory relationship as well as the value placed on CS by occupational therapy supervisees. A further Australian study [20] of allied health professionals used focus groups and individual semi-structured interviews to explore participant experiences in their own words and descriptions. These studies called for further CS research with rural and remote allied health professionals to address existing knowledge gaps. To address these gaps, this study explored the CS practices and experiences of rural and remote allied health professionals using qualitative methodology. This study was part of a larger mixed methods project that investigated the factors that contribute to high quality CS in allied health.

## Methods

### Ethics

Ethics approval for this study was obtained from the Darling Downs Hospital and Health Service Human Research Ethics Committee for multi-sites [Ref: HREC/16/QTDD/22] and the University of South Australia Human Research Ethics Committee [Ref: 0000035268]. Following this all required site specific approvals were obtained.

### Design

This study employed a qualitative research design that used individual, semi-structured interviews. Semi-structured interviews utilise a loose structure consisting of open-ended questions that allow the interviewer and interviewee to pursue an idea or response in more detail [21].

### Setting and participants

The study was undertaken in two Australian states, namely Queensland and South Australia. They were chosen as they have comparable supervision frameworks for allied health [22, 23]; and due to their geographical spread, both states have a dispersed non-metropolitan population serviced by dispersed allied health professionals. As per these supervision frameworks, all allied health professionals are expected to undertake CS or another form of professional support like mentoring. The frequency and duration of CS health professionals are expected to receive is dependent on their work experience and role. CS sessions are documented by the supervisee and/or the supervisor and these documents are used to guide subsequent sessions. The ratio of supervisor to supervisee in both states is variable and is dependent on the position level, workload, local procedures and supervision culture.

Given the rural and remote focus of this study, participants from all rural and remote facilities in Queensland and South Australian public health services (five Queensland Health rural and remote Health Service Districts and Country Health South Australia Local Health Network) were invited to participate in this study. Although participants invited were from physiotherapy, occupational therapy, speech pathology, psychology, podiatry, dietetics, exercise physiology and social work, the interviewees recruited were from six of these professions (see Table 1). These disciplines were chosen due to similarities in CS practices between them, as well as in line with other similar studies in this field [16, 18].

**Table 1:** Participant demographics and information about CS received.

Demographic Characteristics	Number (total n = 16)
**Gender**	
Male	1
Female	15
**Workplace location**	
Queensland	11
South Australia	5
**Nature of role**	
Predominantly clinical	10
Predominantly leadership	6
**Profession**	
Dietetics	4
Social work	3
Speech pathology	3
Occupational therapy	3
Physiotherapy	2
Podiatry	1
*Position level	
Junior	1
Intermediate	9
Senior	6
**Mode of CS**	
Face-to-face	2
Telesupervision	14

* Junior positions are primarily responsible for clinical work and are targeted at new graduates. Intermediate positions are occupied by health professionals with some work experience and these roles involve some non-clinical duties such as clinical education, providing supervision etc. Senior positions are targeted at those with more work experience and these roles have leadership, management, research and/or education responsibilities and may have a small clinical component.

### Procedure

Participants were supervisees and were recruited to this study using a purposive maximum variation sampling strategy [24–26]. The allied health rural leaders in both states were identified through key allied health contacts and all were subsequently contacted to obtain a list of eligible participants. The first author then sent out email invitations to 56 allied health professionals from a broad range of experience, nature of their role and years of experience to capture diverse variations. Interested participants provided consent and participated in individual in-depth, semi-structured interviews. Although some interviewees also provided supervision to other staff, for the purpose of this study they were asked to discuss their perspectives on the supervision they receive.

An interview guide was developed based on the literature and previous studies conducted by the authors [11–13]. Some broad topics (framed as questions) were identified by the authors to help uncover the participant’s meaning or perspective [27, 28] The topic categories included supervision characteristics, supervisory relationship, rural and remote supervision (enablers, barriers and recommendation) and telesupervision. The first author that conducted the interviews allowed the participants to frame and structure their responses. The questions posed were open-ended questions followed by requests for elaboration [27, 29]. All the interviews were conducted via telephone and were recorded with permission. Interviews were conducted until no new information was obtained.

### Data analysis

Data analysis was undertaken iteratively and data were transcribed verbatim by an independent typist. The data were managed using the NVivo software ^TM^ [version 10], and were subsequently subjected to inductive content analysis. Content analysis is a research method for the subjective interpretation of the content of the text data through the systematic classification process of coding and identifying themes or patterns [30, 31]. Content analysis allows the researcher to measure the frequency of different categories and themes [32, 33, 34, 35].

Numerous strategies were employed to ensure the research rigour of the qualitative data analysis and interpretation processes. Strategies used to promote credibility, transferability, dependability and confirmability of the analyses and interpretation processes included adherence to semi-structured interview guide, audiotaping interviews, transcribing verbatim by an independent typist, coding by the first two authors, cross checking between coders and member checking of a proportion of data [15, 29, 36, 37]. All disagreements between the coders were resolved through further discussions.

## Results

Sixteen out of seventeen allied health professionals that made contact, consented and completed the interviews. Participants were from rural and remote sites representing a broad range of clinical and rural experience, and from dietetics, social work, speech pathology, occupational therapy, physiotherapy and podiatry. All participants were in one-to-one supervision relationship, with most interviewees also receiving other additional forms of professional support like mentoring, peer supervision and other informal forms of professional support. More than half the interviewees reported choosing their current supervisor, whilst the rest had their supervisors allocated to them. Frequency of supervision sessions received ranged from fortnightly to bi-monthly, with sessions lasting for at least 60 minutes. Duration in current work role ranged from four weeks to nine years. Four of the sixteen participants were transitioning from existing clinical supervision arrangements and entering or seeking new supervisory relationships. The interviews lasted from 28 to 55 minutes. Further information about the participants and CS characteristics has been presented in Table 1.

Content analysis of the interview data resulted in the following eight themes:

### Value of CS in rural and remote areas

All participants described the value and benefit of CS in their roles. Participants stated that rural and remote roles can be isolating and resilience building through CS is integral to working in such roles. In this context, they reported CS as a valuable opportunity to network with colleagues, find answers to questions, validate clinical practice and feel supported in their roles. Most participants emphasised that this is different for supervisees in metropolitan areas where there is easy access to other professionals to draw support from. One participant, a social worker with over 20 years of rural work experience said:

“…I find CS very nourishing, and the lady I work with (supervisor) has such grace and humour that it’s a truly enjoyable experience. Even if you’re working through some particularly difficult issues it’s a really enjoyable experience…the feedback you get is really affirming and it’s like you’re on the right track…. I think that’s very very helpful” (Interviewee 1, female)

One participant that had clinical governance responsibility for around 100 physiotherapists said:

“I think in terms of it (CS) being a strong part of a recruitment and retention strategy it’s really important. We have a lot of staff who are either sole practitioners or work within small teams of maybe just two or three. So CS is a really valuable tool so that they continue to feel supported….” (Interviewee 4, female)

### More than one form of professional support

A predominant theme arising from the interviews was that most participants were linked into a number of professional support arrangements on top of one-to-one CS. These include mentoring, peer group supervision, peer networks and informal/ad hoc arrangements. It appears that just one form of professional support (e.g., one-to-one CS) may not be adequate to meet the broad range of learning needs inherent to rural generalist roles. One participant remarked:

“I actually think it is really beneficial. I actually did more intensive supervision with a mentor as well as a clinical supervisor, as well as another supervisor–like I had three supervisors when I first came out to this area–but I found it really good and it’s really good to make contacts ….” (Interviewee 7, female)

### Impact of contextual factors

It was clear from the interviews that supervisees considered it essential for supervisors to have a good understanding and/or experience of working in a rural/ remote context. This appeared to be as important and desirable as supervisors’ knowledge and clinical experience. Often, rural and remote allied health professionals tend to be sole practitioners, have a broad range of caseload and spend considerable time travelling to smaller sites. This appeared to create a need for clinical and professional advice that is unique and relevant to this context. A participant that was receiving CS from a supervisor that worked in a nearby bigger centre had this to say:

“I think the fact that their (supervisor’s) department is so so different to this health service. So I think their expectations are very mismatched with what services are actually like on the ground in such a remote area. I know that they are only a couple of hours by plane, but they may well be a million miles away….the recommendations or suggestions that she provides me don’t necessarily solve the problem because of the context” (Interviewee 12, female)

One participant that was receiving CS from a supervisor from another health service described the difference in the work contexts:

“I might spend twelve hours one day at work and four-five hours of that is travelling to get to do treatments to then go home again. So whilst you are in a bigger center and you are based in one site, you’re not having as much moving around, you’re not having to plan, spend as much time planning your travel and lists and everything like that “(Interviewee 13, female)

### Limitations of remote CS

Key content areas covered in CS included normative, restorative and formative aspects as per the Proctor’s model. However, given most participants used technology for CS, there appeared to be an increased emphasis on normative and restorative aspects, limiting engagement in formative or technical aspects of CS. On the ground, this appears to result in lack of emphasis on observing patient care events, practicing a skill or reviewing patient chart notes.

One participant said:

“So we spend most of our time discussing service development and what processes and procedures happening in her (supervisor’s) unit that I may be able to implement here. Really, I spend a lot of time debriefing about the challenges of working in such a remote area, and yes, they are the main sorts of things” (Interviewee 12, female)

A participant thus described the limitation of formative aspects in telesupervision:

“It’s one thing to describe scenarios and that over the phone or videoconference, but it’s quite different if the person is sitting with you and you can show them or be part of that physically….” (Interviewee 4, female)

Another participant employed in a senior leadership role, whilst commenting on this issue said:

“Yes absolutely that the ability to not be able to follow or respond to–like to go and have a look at a client on the ward or to watch or observe an education session. I think that hinders the depth that can be achieved with CS, and the usefulness too….” (Interviewee 2, female)

### Optimising the benefits of telesupervision

It appears that telesupervision can work well if some conditions are met including supervisee and supervisor preparedness for the CS session, augmentation with occasional face-to-face sessions and working with a supervisor that the supervisee knew prior to entering the CS relationship. Participants reported that telesupervision, despite the technological barriers, can be beneficial to undertake. Multiple participants reiterated the importance of planning telesupervision in advance to avail videoconference and room facilities. Some participants said:

“The fact that I met my supervisor in person when I commenced really helped us establish a relationship so when we transitioned to using the telephone it wasn’t such a problem” (Interviewee 12, female)

“…your preparation (in telesupervision) is kind of vital…when it is on the phone and it’s very allocated time, so to try and get the most out of your time that you have. So I find telephone supervision to be at times even better than face-to-face because of that prior preparation that you can get your thoughts organised…” (Interviewee 5, female)

### Organisational factors can be an enabler and a barrier

Some participants reported having CS frameworks and procedures in place which promoted engagement in CS. Many comments were made about the usefulness of having a CS framework organization-wide, associated resources and training to up-skill allied health professionals in CS. However, some participants commented on the need for reviewing the framework in place and provision of another round of CS training for supervisees and supervisors, given the high staff turnover rates in rural and remote settings. One participant, an experienced occupational therapist in a leadership role said thus:

“We have our clinical supervision framework, which also has supervision tools available to staff such as activity logs, records, learning style questionnaires…I feel that they are very beneficial for new clinicians coming through to help them develop their skills and build on their experience with uni. So those things I think really support new grads…” (Interviewee 5, female)

On the other hand, some participants reported to having organisational barriers that reduced CS engagement and efficiency. Specifically, being operationally managed by someone different from their own profession appeared to hinder CS engagement and outcomes. Furthermore, participants expressed the desire for closer alignment of CS and operational management.

One participant said:

“..the problem is that maybe management is not understanding or even if you are in allied health sitting under a nurse not understanding the recommendations from the organisation around supervision, it would be quite easy for you to never have supervision because it is not managed and there is no governance around it…” (Interviewee 15, female)

One participant commented on the mis-alignment of policy makers in larger metropolitan centres and those engaging in CS in rural and remote locations:

“When the State level policies and everything made by the practice what is currently governed and all the policy officers and everyone are sitting in the metropolitan centre, but it is the real underground clinicians have the challenges” (Interviewee 14, male)

### Need for assessment/measurement in CS

Many participants reported the need for CS audits and for examining the currency of CS frameworks and models used by the organisation. Some participants highlighted the need to roll out training regularly given the transient workforce in rural and remote areas. One participant said:

“…we’ve got a model in place that’s probably been a fairly stagnant model for a while. So it would be nice to have it reviewed. The agreements haven’t changed in the past six or seven years. So I think a potential recommendation might be just reviewing that and making sure that it is still meeting the needs of the organisation” (Interviewee 4, female)

Another participant, an experienced occupational therapist in a team leader role, when discussing how CS experience can be made better for her team members, said:

“….if there is some way of assessing the quality of the supervision, and I think giving our clinicians an opportunity to feedback to us around supervision…” (Interviewee 11, female)

### Unique characteristics of rural and remote CS

Various unique characteristics of CS became apparent through the interviews. Some participants considered friendship and social aspects of CS crucial in the supervisory relationship.

One participant who reported a positive supervisory relationship with her supervisor said:

“I guess when we have supervision we usually go for coffee. We usually catch up with what’s happening in each other’s lives and then we talk about business…” (Interviewee 1, female)

Many participants reported on drawing professional support from the multi-disciplinary team and engaging in informal, interprofessional CS. One participant, who had recently moved from a metropolitan centre to a remote town said:

“I have had a few decent conversations with some of the other professionals about how they manage their time, how they manage to see clients from rural and remote areas—what strategies they use–when they use face-to-face or tele-health. So, not in a formal setting but definitely I’ve had a number of conversations with other members of the team informally” (Interviewee 9, female)

Participants reported that the eligible pool of supervisors in rural and remote areas was limited. Participants also highlighted the difficulty in finding time for CS. However, those participants that planned CS sessions ahead of time and saw CS as a priority were more successful in engaging with CS. The majority of participants reported about the flexible nature of CS they received and saw it as an essential characteristic of rural and remote CS. One participant while commenting on flexibility said:

“I think rurally you need that flexibility and I think sometimes it’s even good to have two supervisors because it’s very difficult to get a supervisor who can meet all of your needs. If you have a supervisor and a mentor maybe that cover two different areas I think that would be really nice….” (Interviewee 9, female)

## Discussion

This study was undertaken to further the evidence for CS of rural and remote allied health professionals. As this study recruited participants from two Australian states and from six allied health professions, it provides rich, information on the factors that improve the quality of CS for rural and remote health professionals. As rural and remote CS has not been adequately researched to date, the findings from this study are expected to contribute to the emerging evidence base in this area. Furthermore, as this study explored individual participant perspectives, evidence is now available on what happens at the coalface in the Australian context.

The predominant finding of this study is the value placed on CS by rural and remote health professionals. Whilst CS has been shown to benefit health professionals in all geographical and practice contexts [4, 11, 2], its value is more pronounced in rural and remote healthcare settings. These areas often face workforce challenges such as recruitment, retention and dealing with a transient workforce [38, 39]. Most rural roles in Australia are also generalist roles where the scope of clinical practice is broad and varied. Health professionals practicing rurally are also known to face professional isolation, challenging cultural contexts and lack of resources [14]. These issues enhance the value of CS for this under-resourced group. This is further highlighted by the finding that more than one form of professional support is utilised by most study participants. This finding is expected to reinforce to employing organisations, the unique professional support needs of rural and remote health professionals. This builds on findings from previous studies that a ‘one size fits all’ approach does not work for CS [20,40].

A key finding from this study was the role context played in CS. Participants that were supervised by those that had previously worked in a rural context found their CS arrangements more useful and relevant. This reiterates the importance of achieving a good supervisor-supervisee fit that has been highlighted in previous studies [13, 15]. It is important to take into consideration prior experience of the supervisor in the initial phases. A systematic review of telesupervision studies found that supervisors with rural experience achieved positive CS outcomes for rural supervisees [40]. Where this is not possible, supervisors can be encouraged to complete site visits of the supervisee’s work place to understand the context better. Furthermore, the findings of this study have highlighted the challenges supervisees face when reporting to someone from another profession or stream. As this is common in rural and remote regions in countries such as Australia given smaller number of health professionals, guidelines need to be established on managing such partnerships.

This study revealed that supervisees embraced the restorative and normative functions of CS more than the formative functions when they used technology for CS. Proctor’s model emphasises that all the three functions are essential in CS [10]. Whilst the challenges of engaging in ‘hands on’ demonstration or practice sessions in telesupervision are real, it is imperative that this is acknowledged as a barrier and working solutions are found. Failure to address this proactively may pose a risk for clinical governance. This is especially essential for new graduates, professionals that are new in their roles and those that have changed practice areas.

It is already well-acknowledged that organisational factors can be a barrier to high quality CS [15, 20]. This study has added evidence to this and highlighted the need for organisations to ensure currency of their CS frameworks, tools and trainings. Additionally, this study has highlighted the need for evaluation of CS practices in rural and remote areas. Interestingly time for CS was again cited as a barrier to CS consistent with previous studies [15, 20]. However, multiple participants in this study made it clear that when CS is treated as priority and when it is planned in advance, this barrier can be overcome. Given the value of CS to rural and remote health professionals, it is imperative to prioritise and quarantine CS time.

It is best to bear in mind the type of CS this paper describes when interpreting the study results given the varied understanding of CS and the confusion about the different CS terminologies internationally [41, 42].

### Limitations

This study explored the perspectives of supervisees about the factors that contribute to high quality CS. Similar studies with supervisors are required to further the evidence in this area. Studies are also required that would demonstrate the impact of CS on patient outcomes. All interviews were conducted via telephone as participants were recruited from a number of rural and remote sites in two Australian states. As non-verbal communication is unavailable to the interviewer whilst using the telephone, there is a risk that not all information conveyed by participants were captured. However, the first author is experienced with conducting telephone interviews and employed a number of strategies [such as pausing adequately, using clarifying questions and disciplined turn taking] to ensure that information was efficiently captured. Furthermore, 15 of the 16 participants in this research were female and as such it is unknown what, if any, different perspectives may have been captured if there were more male participants. The over-representation of female participants, however, reflects the gender divide that is commonly seen amongst allied health professions in rural and remote settings.

### Conclusions

This is the first known study to explore rural and remote allied health supervisee perspectives on CS quality. Rural and remote areas are known to be underserved and health professionals in those regions often experience professional isolation. As this group is also known to experience unique challenges in healthcare delivery, specifically targeted studies were required. This study addressed this gap and highlighted a number of themes related to CS quality in allied health supervisees. Results reinforced that the benefits of CS are more-pronounced rurally and shed light on the crucial role CS played in recruitment and retention of the rural health workforce. These findings are expected to add further evidence to the growing evidence of the impact of CS on healthcare. The findings from this study also provide important lessons for employing organisations about the unique context of CS in rural and remote regions.

## Abbreviations

CS: Clinical Supervision
